# VNTR polymorphism in the breakpoint region of *ABL1* and susceptibility to bladder cancer

**DOI:** 10.1186/s12920-021-00968-1

**Published:** 2021-05-05

**Authors:** Min-Hye Kim, Gi-Eun Yang, Mi-So Jeong, Jeong-Yeon Mun, Sang-Yeop Lee, Jong-Kil Nam, Yung Hyun Choi, Tae Nam Kim, Sun-Hee Leem

**Affiliations:** 1 Department of Biomedical Sciences, Dong-A University, Busan, 49315 Korea; 2 Department of Health Sciences, The Graduated of Dong-A University, Busan, 49315 Korea; 3 Research Center for Bioconvergence Analysis, Korea Basic Science Institute, Ochang, 28119 Korea; 4 Department of Urology, Research Institute for Convergence of Biomedical Science and Technology, Pusan National University Yangsan Hospital, Yangsan, 50612 Korea; 5 Department of Biochemistry, College of Oriental Medicine, Anti-Aging Research Center, Dong-Eui University, Busan, 47227 Korea; 6 Department of Urology, Medical Research Institute, Pusan National University Hospital, Busan, 49241 Korea

**Keywords:** *ABL1*, Breakpoint cluster region, VNTR, Polymorphism, Bladder cancer

## Abstract

**Background:**

*ABL1* is primarily known as a leukemia-related oncogene due to translocation, but about 2.2% of *ABL1* mutations have been identified in bladder cancer, and high expression in solid cancer has also been detected.

**Methods:**

Here, we used the NCBI database, UCSC genome browser gateway and Tandem repeat finder program to investigate the structural characterization of the *ABL1* breakpoint region and to identify the variable number of tandem repeats (VNTR). To investigate the relationship between *ABL1*-MS1 and bladder cancer, a case-controlled study was conducted in 207 controls and 197 bladder cancer patients. We also examined the level of transcription of the reporter gene driven by the *ABL1* promoter to determine if the VNTR region affects gene expression.

**Results:**

In our study, one VNTR was identified in the breakpoint region, the intron 1 region of *ABL1*, and was named *ABL1*-MS1. In the control group, only two common alleles (TR13, TR15) were detected, but an additional two rare alleles (TR14, TR16) were detected in bladder cancer. A statistically significant association was identified between the rare *ABL1*-MS1 allele and bladder cancer risk: *P* = 0.013. Investigating the level of transcription of the reporter gene driven by the *ABL1* promoter, VNTR showed inhibition of *ABL1* expression in non-cancer cells 293 T, but not in bladder cancer cells. In addition, *ABL1*-MS1 was accurately passed on to offspring according to Mendelian inheritance through meiosis.

**Conclusions:**

Therefore, the *ABL1*-MS1 region can affect *ABL1* expression of bladder cancer. This study provides that *ABL1*-MS1 can be used as a DNA fingerprinting marker. In addition, rare allele detection can predict susceptibility to bladder cancer.

**Supplementary Information:**

The online version contains supplementary material available at 10.1186/s12920-021-00968-1.

## Background

The Abelson murine leukemia virus oncogene homolog 1 (*ABL1*) encodes a kinase and is activated by several switch kinases such as receptor tyrosine kinase (*RTK*) and cyclic AMP (*cAMP*), and is involved in various cellular processes including cell growth, division, differentiation and migration [1]. *ABL1* presents at 9q34.12 on chromosome 9, translocate with the breakpoint cluster region (BCR) gene on chromosome 22 to form the Philadelphia chromosome, the cause of chronic myelogenous leukemia [2]. The BCR region present in *ABL1* contains retrotransposon repeats, a DNA sequence that can be rearranged within the genome, and satellite DNA, a short repeat sequence. It is known that the high density of these repeated sequences can lead to disease-causing genomic structural instability [3, 4]. Although high expression occurs mainly in chronic myelogenous leukemia by translocation of *ABL1*, it has recently been reported that the *ABL1* gene may have an effect on the onset of solid cancer. In particular, an association with bladder cancer in the BCR region of *ABL1* has also been reported [5–8].

Bladder cancer is largely divided into non-muscle invasive bladder cancer (NMIBC) and muscle invasive bladder cancer (MIBC) [9]. At the time of bladder cancer diagnosis, about 70% are NMIBC, and 10% of patients have already found metastatic bladder cancer that has spread to other organs. It often occurs in elderly people in their 60 s and 70 s, and there are three to four times more male patients than females [10]. In addition, smokers are known to have a very high risk of bladder cancer. While non-muscle invasive bladder cancer patients have a 5-year survival rate of 75%, those who have suffered once are very likely to recur [11]. Previous studies have confirmed that over 80% of *ABL1* is expressed in MIBC through IHC experiments, and that imatinib, which inhibits the *BCR-ABL1* gene from promoting tyrosine kinase action in cancer cell membranes, may be useful in these patients [12]. And it has been reported that about 2.2% of mutations in the *ABL1* are found, especially in bladder cancer patients [13]. In addition, the *ABL1* region at position 9q34.12 on chromosome 9 has also been reported as a loss of heterozygosity (LOH) marker that may affect the recurrence of bladder cancer [14].

The occurrence of LOH in genomic DNA has been reported in many cases due to the instability of the repeat sequence [15]. Reports of LOH for this region in bladder cancer suspect a repeat sequence region associated with the occurrence of cancer [14]. Although the function of the repeat sequence in the genome has not been identified much, it is known to be related to genome evolution [16], and its association with disease has also been reported [17]. Many retrotransposon repeats and simple sequence length polymorphisms (SSLP) were identified in the BCR region of *ABL1*, and one VNTR in the *ABL1* intron 1 belonging to the BCR region was identified in this study. The VNTR belonging to SSLP can be used as a DNA typing marker. Thus, it can be used as a paternity marker and is also used as a susceptibility marker for diseases such as Huntington's chorea and cancer [18–20]. Although there were reports of structural features in the BCR region of *ABL1*, VNTR was not reported in previous studies, so this study focused on the association between VNTR in the BCR region and bladder cancer.

First, whether the *ABL1*-MS1 allele is polymorphic was investigated, and a case–control study was conducted using genomic DNA samples from male controls and male bladder cancer patients. In addition, family genomic DNA samples were used to confirm whether this region could be used as a paternity marker. We also investigated the effect of the *ABL1*-MS1 allele on gene expression identified in control and bladder cancer patients. The functional potential of *ABL1*-MS1, according to the results, will be discussed in this paper.

## Methods

### Analysis of *ABL1* genomic structure and primer construction for VNTR

The NCBI database (https://www.ncbi.nlm.nih.gov/gene) and the UCSC genome browser gateway (https://genome.ucsc.edu/cbi-bin/hgGateway) were used to analyze the genome structure of the BCR region in *ABL1*. The repeat regions were also analyzed using the RepeatMasker (www.repeatmasker.org) program and the Tandem repeat finder program (http://www.tandem.bu.edu/trf/trf.html). For selecting the VNTR region among SSLP, we identified a VNTR with a repeat unit length of 10–100 bp and a program algorithm score of 1000 or more. The primers’ construction for amplifying the selected VNTR region was made using the Primer 3 program (http://www.bioinfo.ut.ee/primer3-0.4.0/) and the Primer-BLAST program (http://www.ncbi.nlm.nih.gov/tools/primer-blast). Primer sequences used in this study are as follows: *ABL1*-MS1 primers (Forward 5′-GGAATGGAAGGGGTGTTGGG-3′ and Reverse 5′-ACCCACTTCTCCACCTCCTC-3′).

### Preparation of genomic DNA from peripheral blood lymphocytes of controls and bladder cancer patients

Genomic DNAs were extracted from peripheral blood lymphocytes of 207 male controls and 197 male bladder cancer patients to confirm the polymorphism of the *ABL1*-MS1 region. A total of 207 individuals in the control group with no personal history of cancers or current cancer were recruited and completed an interview. The controls consist of similar proportions of sex and age range to the cases (Table 1). Cases with bladder cancer and controls were recruited from two different hospitals in the same city. The bioethics committees of Dong-A University Hospital, Pusan National University Hospital approved research plan and procedure: [Dong-A University Hospital (#IRB-07-10-7; Busan, Korea), Pusan National University Hospital (#IRB-H-1706-002-007; #IRB-H-1804-002-065; Busan, Korea)]. For PCR (polymerase chain reaction) experiments, genomic DNA was isolated from 400 µL of whole blood, using the Blood and Cell Culture DNA Mini Kit (Qiagen, CA, USA).

**Table 1:** Age distribution of controls and bladder cancer cases

Age (years)	Controls *N* (%)	Bladder cancer *N* (%)	*P* value
40–49	10 (4.8)	7 (3.6)	0.9974
50–59	40 (19.3)	39 (19.8)	
60–69	91 (44.0)	91 (46.2)	
70–79	56 (27.1)	50 (25.4)	
80–89	10 (4.8)	10 (5.1)	
Total *N*	207	197	
Average	65.2	65.7	
Median	66	66	

### PCR analysis of *ABL1*-MS1

PCR analysis of human DNA samples was performed using the Coregen Taq polymerase (Coregen, Busan, Korea) with 100 ng genomic DNA. Genomic DNA was amplified using primers (forward and reverse) in addition to a standard PCR mixture of 50 mM KCl, 10 mM Tris–HCl (pH 9.0), 3 mM MgCl_2_ and 0.2 mM dTTP, dCTP, dGTP and dATP, made up to a final volume of 30 µl. PCR was conducted in a 9700 Thermalcycler (Perkin-Elmer Inc, CA, USA) under the following conditions: 94 °C for 2 min, 30 cycles of 94 °C for 30 s, 68 °C for 20 s, 72 °C for 3 min and a final extension step at 72 °C for 7 min. PCR products were visualized by 2% SeaKem LE agarose (Lonza, Basel, Swiss) ethidium bromide gel electrophoresis in TAE buffer at 60 V for 16 h.

### Plasmid construction

To generate a luciferase reporter vector having a fragment of the *ABL1* 5'-promoter region, a fragment was amplified from normal male genomic DNA containing the *ABL1* genomic sequence by PCR and inserted into the *Nhe*I/*Hind*III site of the luciferase reporter vector pGL3-Basic (Promega, Madison, WI, USA). To confirm the effect of *ABL1*-MS1 on *ABL1* expression, two common (TR13 and TR15) alleles and two rare alleles (TR14 and TR16) were amplified from genomic DNA derived from control and case and inserted into the *Hpa*I/*Sal*I sites of the p2000 fragment to generate the reporter plasmids p2000 + TR13, p2000 + TR14, p2000 + TR15, and p2000 + TR16 (Fig. 4) All constructs were confirmed by DNA sequencing.

### Cell lines and luciferase assays

The following human cell lines were tested for the effect of *ABL1*-MS1 on *ABL1* expression: 293 T (human embryonic kidney cell line obtained from the Korean Cell Line Bank; KCLB, Seoul, Korea) and UM-UC3 (bladder cancer cell line). For the luciferase assay, cells (4 × 10^4^) were seeded in 24-well plates, cultured overnight and transfected with the *ABL1* promoter-luciferase plasmids (0.3 μg per well) using the jetPrime transfection reagent (Polyplus-transfection Inc., NY, USA) at a ratio of DNA/jetPrime of 1:3. Analysis of the cells was performed using a dual-luciferase reporter assay system (Promega) 24 h after completion of the transfection procedure. Firefly luciferase activities were normalized to Renilla luciferase activity, and activity was expressed in relative luciferase units to reflect the promoter activity. Triplicate transfections of each construct were tested for each experiment, and the final results were calculated from four independent experiments.

### Phylogenic tree construction

Nucleotide diversities of each repeat unit of the *ABL1*-MS1 were calculated for each minisatellite. To examine the extent of sequence divergence, the pairwise distance was calculated using a modified Kimura’s two-parameter model with MEGA3 software [21, 22]. We obtained neighbor-joining trees [23] with Kimura’s two-parameter model using Clustal W [24].

### Statistical analysis

Regression analysis was used to determine odd ratios (ORs) for the association between alleles of the VNTR region and the risk of bladder cancer incidence among the control group and bladder cancer patient groups. 95% confidence intervals (CIs) for OR were calculated using logarithms of OR and standard error (SE). The difference was considered to be CI 95% significant. The data were examined using R statistical software (https://www.socscistatistics.com/tests/fisher/default2.aspx) with the Fisher exact test [25]. All tests were two-sided, with *P* < 0.05 considered statistically significant.

## Results

### Genomic analysis of *ABL1*-breakpoint cluster and identification of VNTR

In order to find out the distribution of repeat sequences for the *ABL1* BCR region, which is known to be a very important region for disease development, we investigated through the Repeatmasker (http://www.repeatmasker.org/) and UCSC genome browser gateway database (http://genome.ucsc.edu/cgi-bin/hgGateway?org=human). (Additional file 1: Fig. S1). In the *ABL1* BCR region of about 142 kb in length, short interspersed nuclear element (SINE) was present at the highest frequency, and many repetitive sequences such as long interspersed nuclear element (LINE), long terminal repeat (LTR), and simple repeats were identified (Additional file 1: Fig. S1).

**Fig. 1:**
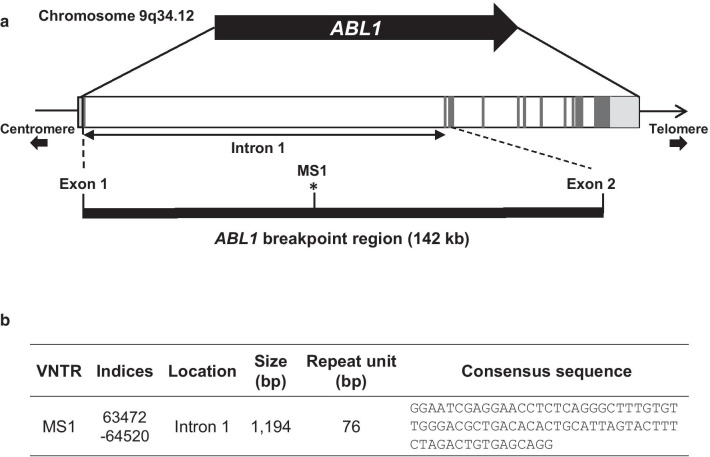
VNTR analysis of *ABL1*-breakpoint cluster region. **a** Schematic of the VNTR region of *ABL1*. 11 exons are marked as black boxes and 10 introns as white boxes. One VNTR region was identified in the *ABL1*-breakpoint cluster region, named MS1, and marked with an asterisk. **b** The location of *ABL1*-MS1, the size of the repeating unit, and the consensus sequence were confirmed from the genome information of NCBI

*ABL1* consists of 11 exons and 10 introns, approximately 173 kb in length, located on chromosome 9q34.12 (Fig. 1a). Using the Tandem repeat finder program (http://tandem.bu.edu/trf/trf.html), an area within intron 1 that belongs to the BCR area was analyzed, and one VNTR area (algorithm score > 1000) was identified. We named it *ABL1*-MS1 and the repeat unit of this VNTR was 76 bp, and the expected PCR product size was 1194 bp (Fig. 1b). Through analysis of the GenBank database using the BLASTN program, no significant similarity was found between the *ABL1*-MS1 region and the previously identified region. Therefore, *ABL1*-MS1 identified in this study is unique to *ABL1*, and the properties they impart may be directly related to the *ABL1* function.

### Identification of polymorphism of *ABL1*-MS1 and association of rare alleles of *ABL1*-MS1 with bladder cancer

It is known that the expression of *ABL1* is high in solid cancers including bladder cancer [26–28], and the structural characteristics of *ABL1* are related to the expression [29]. It has also been reported that the LOH of *ABL1* can be used as a diagnostic marker for bladder cancer recurrence [14]. Therefore, we tried to investigate the possibility whether the variant alleles of the VNTR site identified in this study could be used as a predictive diagnostic marker for bladder cancer. To determine whether the variant alleles of *ABL1*-MS1 could affect the susceptibility of bladder cancer, we examined the frequency of the *ABL1*-MS1 allele and its association with bladder cancer.

**Fig. 2:**
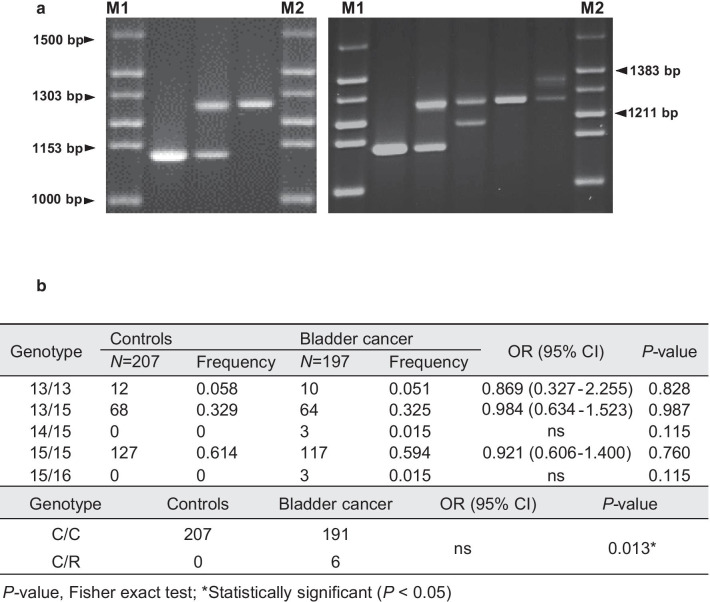
Allelic type patterns of *ABL1*-MS1 in cancer-free male controls and bladder cancer patients. **a** Haplotype patterns in cancer-free controls and cases with bladder cancer. The left panel is the haplotype pattern for *ABL1*-MS1 region from control samples. Three genotypes were identified consisting of two different *ABL1*-MS1 alleles. The right panel is the *ABL1*-MS1 haplotype pattern seen in bladder cancer patients, showing five genotypes consisting of four different *ABL1*-MS1 alleles. The first and last lanes correspond to a 100-bp (M1; Invitrogen Co., CA, USA) and a 1-kb size marker (M2; Invitrogen Co.). **b** Frequency of genotypes between controls and bladder cancer cases. N corresponds to the total number of samples tested for the allele of *ABL1*-MS1. C corresponds to the common alleles (13TR, 15TR) and indicates alleles with a frequency of 1% or more. Rare alleles (R) with a frequency of less than 1% correspond to 14TR and 16TR. * Statistically significant (*P* < 0.05)

To confirm the polymorphism of *ABL1*-MS1, a cases-controls study was performed using genomic DNA from 207 cancer-free male controls and 197 male bladder cancer patients (Fig. 2). Table 1 shows the distribution and ratio by age for the control group and the patient group with bladder cancer used in this study. There was no significant difference between groups according to age (*P* = 0.997).

Polymorphic alleles in controls were observed in *ABL1*-MS1 with two alleles containing 13- or 15-repeats, 1113 bp and 1265 bp in length (Table 2). In contrast, four alleles were identified in the bladder cancer patient group, including two rare alleles [the number of repeats was 14 (1189 bp) or 16 (1341 bp); the expected frequency for rare alleles was set as < 1%] and two common alleles (the number of repeats was 13 or 15; Table 2). Rare alleles of *ABL1*-MS1 were identified only in bladder cancer, and the most common alleles had 15 repetitions. The heterozygosity of these two groups was 0.345 in the controls and 0.359 in the patient’s group with bladder cancer, with no significant difference.

**Table 2:** Comparison of allelic frequency of *ABL1*-MS1 between controls and bladder cancer

# Repeat	Controls		Bladder cancer		OR (95% CI)	*P* value
	*N* = 414	Frequency	*N* = 394	Frequency		
13 (1113 bp)	92	0.222	84	0.213	0.948 (0.668–1.343)	0.798
14 (1189 bp)	0	0	3	0.008	ns	0.116
15 (1265 bp)	322	0.778	304	0.772	0.965 (0.684–1.362)	0.866
16 (1341 bp)	0	0	3	0.008	ns	0.116

Alleles	Controls	Bladder cancer	OR (95% CI)	*P* value
Common alleles (13, 15)	414	388	ns	0.013*
Rare alleles (14, 16)	0	6		

*P *value, Fisher exact test

*Statistically significant (*P* < 0.05)

When examining the haploid genotype of *ABL1*-MS1, three patterns were found in the control group: genotype containing two of TR13, two of TR15 or each of TR13 and TR15. In bladder cancer, five different genotypes were identified as indicated in the right panel of Fig. 2a. There were no statistically significant differences in the frequency of each genotype in the two groups (Fig. 2b). The frequency of appearance of common alleles or rare alleles of *ABL1*-MS1 was compared and analyzed in control and bladder cancer, respectively. The total frequency of rare *ABL1*-MS1 alleles was 1.5% in cases, compared to 0% in cancer-free controls (Table 2). The incidence of rare alleles in the bladder cancer patient group was statistically significant compared with the control group (*P* = 0.013; Table 2). In addition, the frequency of incidence of C/R genotypes with rare alleles also showed statistically significant values (*P* = 0.013) when comparing the bladder cancer group and the control group (Fig. 2b). These results indicate that the rare allele of *ABL1*-MS1 is associated with the risk of bladder cancer.

The average age at diagnosis of bladder cancer patients is about 65 years old. Based on this age, a group that showed onset at a younger age can be divided into a young group (< 65 years), and a group diagnosed at an older age can be divided into an old group (≥ 65 years). The frequency of rare alleles was higher in younger cases (2.92%) than in older cases (0.46%) (Table 3). In younger patients compared to elderly patients, the correlation between the rare *ABL1*-MS1 allele and bladder cancer was not statistically significant but showed an increased tendency (6.345, CI 0.73–54.82; *P* = 0.093) (Table 3). Additionally, in comparing the control and cancer patients by the same age group, the association ratio between bladder cancer and the rare *ABL1*-MS1 allele in young patients was also not statistically significant (*P* = 0.061) (Table 3), but the risk tended to increase somewhat.

**Table 3:** Frequency of rare alleles and risk of bladder cancer by age

Age	Controls		Bladder cancer		OR (95% CI)	*P* value
	Common	Rare	Common	Rare		
Younger (< 65)	174	0	171	5	ns	0.061
Older (≥ 65)	240	0	217	1	ns	0.476
Reference (older group)	ns		6.345 (0.73–54.82); *P* = 0.093		OR (95% CI) *P* value	

*P *value, Fisher exact test

*Statistically significant (*P* < 0.05)

**Fig. 3:**
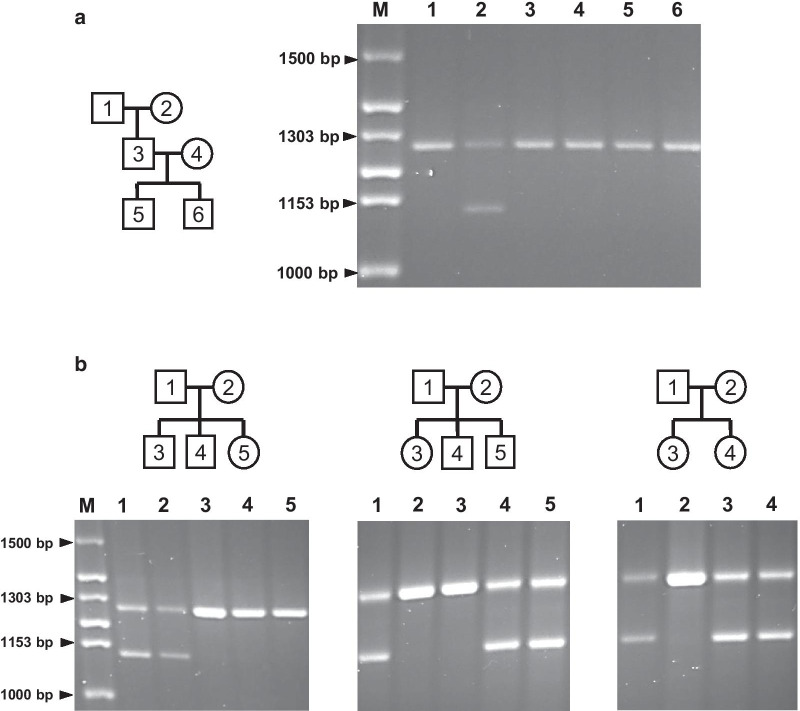
Meiotic segregation of *ABL1*-MS1. **a** Meiotic inheritance of the *ABL1*-MS1 in a third-generation family. *ABL1*-MS1 were analyzed for minisatellite length in genomic DNA from family members. The pedigree demonstrates the relationship between family groups used in this study: first-generation (lanes 1 and 2, grandfather and grandmother, respectively); second-generation (lanes 3 and 4, father and mother); and third-generation (lanes 5 and 6, children from parents 3 and 4. **b** Meiotic inheritance the *ABL1*-MS1 in three of second-generation. The first-generation is denoted as 1 and 2 (mother and father). The second-generation was shown as 3 and 4, and 5 (children). M corresponds to the size marker

To examine the meiotic segregation of *ABL1*-MS1 alleles during meiosis, we selected family groups of two and three generations (four and two families, respectively) (Fig. 3). Genomic DNA was then isolated with peripheral blood lymphocytes from the grandparents, parents and two to three children in each family [30, 31]. Due to the low heterozygosity, two common alleles were detected in *ABL1*-MS1 region in tested families (data not shown), and the transmission of alleles from parent to child in six families was traced (Fig. 3). The results revealed that *ABL1*-MS1 is subject to Mendelian inheritance (i.e., children carried one minisatellite allele from each parent), and new *ABL1*-MS1 alleles were not observed during this analysis.

### Possible function of *ABL1*-MS1 alleles for the expression of *ABL1*

To examine the possible role of minisatellites in intron regions, we cloned, sequenced and aligned the four different alleles (TR13, TR14, TR15 and TR16) of *ABL1*-MS1 (Table 4, Additional file 2: Table S1). The consensus sequence of repeat unit was determined by DNA sequencing of the repeat unit sequence present within the *ABL1*-MS allele, and 20 types of repeat units with a variation of 1–27% were found (Table 4, Additional file 2: Table S1). The consensus sequence for each repeat unit in *ABL1*-MS1 was defined from multiple units within it (Table 4, Additional file 2: Table S1). We next constructed the phylogenic tree for each minisatellite in *ABL1*-MS1, as shown in Additional file 1: Fig. S2a. The distance between the repeat units was analyzed through the degree of divergence in the sequence (Additional file 1: Fig. S2a).

**Table 4:** Composition of repeats unit of *ABL1*-MS1 alleles

Repeat unit	Sequences
Consensus	**GGAATCGAGGAACCTCTCAGGGCTTTGTGTTGGGACGCTGACACACTGCATTAGTACTTTCTAGACTGTGAGCAGG**
MS1-1	GGAATC**A**AGGAACCTCTCAGGGCTTTGTGTTGGGACGCTGACACACTGCATTAGTAC**C**TTCTAGACT**A**TGAGCAGG
MS1-2	GGAATCGAGGAACCTCTCAGGGCTTTGTGTTGGGAC**A**CTGAC**T**CACTGCATTAGTAC**C**TTCTAGACT**A**TGAGCAGG
MS1-3	GGAATCGAGG**G**ACCTCTCAGGGCTTTGTGTTGGGACGCTGACACACTGCATTAGTACTTTCTAGACTGTGAGCAGG
MS1-4	GGAATCGAGGAACCTCTCAGGGCTTTGTGTTGGGACGCTGACACACTGCATTAGTACTTT**G**TAGACTGTGAGCAGG
MS1-5	GGAATCGAGGAACCTCTCAGGGCTTTGTGTTGGGACGCTGACACACTGCATTAGTACTTTCTAGACTGTGAGCAGG
MS1-6	GGAATCGAGGAACCTCTCAGGGCTTTGTGTTGGGAC**A**CTGAC**T**CACTGCATTAGTACTTTCTAGACTGTGAGCAGG
MS1-7	GGAATCGAGGAACCTCTCAGGGCTTTGTGTTGGGACGCTGACACACTGCATTAGTACTTTCTAGACTGTGAGCAG**A**
MS1-8	GGAATCGAGGAACCTCTCAGGGCTTTGTGTTGGGACGCTGACACACTG**T**ATTAGTACTTTCTAGACTGTGAGCAG**A**
MS1-9	GGAATCGAGGAACCTCTCAGGGCTTTGTGTTGGGAC**A**CTGAC**T**CACTGCATTAG**G**ACTTTCTA**A**ACTG**G**GA**A**CAGG
MS1-10	GG**G**ATCGAGGAACCTCTCAGGGCTTTGTGTTGGGACGCTGACACACTGCATTAGTACTTT**G**TAGACTGTGAGCAGG
MS1-11	GGAATCGAGGAACCTCTCAGGGCTTTGTGTTGGGACGCTGACACACTGCATT**G**GTACTTT**G**TAGACTGTGAGCAGG
MS1-12	GGAATCGAGGAACCTCTCAGGGCTTTGTGTTGGGAC**A**CTGAC**T**CACTG**G**ATTAGTACTTTCTAGACTGTGAGCAGG
MS1-13	GGAATCGAGGAACCTCTCAGGGCTTTGTGTTGGGA**AC**C**A**GAC**T**CACTGCATTA**A**TACTTTCTAGACTGTGA**A**CAGG
MS1-14	GGAATCGAGGAACCTCTCAGGGCTTTGTGTTGGGAC**A**CTGAC**T**CACTGCATTAGTAC**CC**TCTAGACT**A**TGAGCAGG
MS1-15	GGAATCGAGGAACCTCTCAGGGCTTTG**C**GTTGGGACGCTGACACACTGCATT**G**GTACTTTCTAGACTGTGAGCAGG
MS1-16	GGAATCGAGGAACCTCTCAGGGCTT**C**GTGTTGGGACGCTGACACACTGC**G**TTAGTACTTTCTAGACTGTGAGCAG**A**
MS1-17	GGAATCGAGGAACCTCTCAGGGCTTTGTGTTGGGACGCTGACACACTGCATTAGTACTTTCTAGACTGTGAG**T**AGG
MS1-18	GGAATCGAGGAACCTCTCAGGGCTTTG**C**GTTGGGACGCTGA**ACAC**CTGCATTAGTACTTTCTA**A**ACTGTGAGCA**AA**
MS1-19	GGAATCGAGGAACCTCTCAGGGCTTTGTGTTGGGA**AC**CTGA**AT**C**C**CTGCATTAG**A**ACTTTC**C**A**A**ACTG**G**GA**A**CAGG
MS1-20	GGAATCGAGGAACCT**T**TCAGGGCTTT**TG**GTTGGGA**AC**CTGAC**CTC**C**C**G**GCA**T**TAA**A**AC**TT**T**T**CA**A**AAC**TG**G**G**A**A**CA**

MS1-TRs	Composition of repeats unit in each minisatellites
MS1-13TR	1–2–3–4–4–5–6–5–4–7–6–6–Δ
MS1-14TR	1–2–3–4–4–8–6–6–5–4–7–6–9–Δ
MS1-15TR	1–2–3–10–11–7–6–6–12–5–4–7–6–13–Δ
MS1-16TR	1–2–14–15–4–4–16–6–6–6–17–4–18–19–20–Δ

Δ: 0.6–0.9 repeat

Then, these four minisatelltes were analyzed using the Transfac software (MATCHTM public version 1.0; http://www.gene-regulation.com/pub/databases.html), which found several putative binding sites for the transcription factor *GR-alpha*, *GR-beta* [32], *EBF* [33], *C/EBPbeta* [34], *NF-1* [35], *FOXP3* [36], *Pax-5* [37, 38], *p53* [39], *STAT4* [40] and *TFII-I* [41] in the repeats of *ABL1*-MS1 (Additional file 1: Fig. S2b). These various transcription factors have been reported to be related to various cancers [42]. Therefore, we speculate whether these transcription factors when associated with *ABL1*-MS1, which play a role in carcinogenesis by influencing *ABL1* activity.

**Fig. 4:**
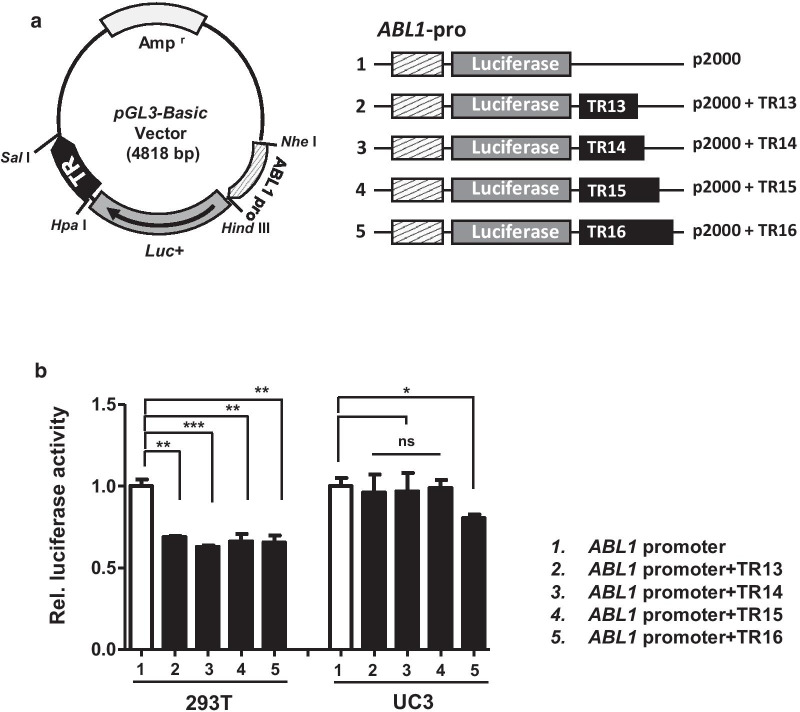
Functional analysis of the VNTRs within *ABL1*-MS1 in luciferase reporter vector promoter region. **a** *ABL1* promoter vector (p2000) and four promoter vectors in which four different lengths of *ABL1*-MS1 (TR13–TR16) were inserted were used. The *ABL1* promoter region is indicated by diagonal squares, and the luciferase gene is indicated by gray squares. Four different lengths of *ABL1*-MS1 were inserted after the luciferase gene and are indicated by black squares. **b** The above five different types of promoter vectors were transfected into 293 T and UC3 cells. *ABL1* promoter activity was measured by luciferase assay

To investigate whether *ABL1*-MS1 affects transcriptional activity, the 2 kb of *ABL1* promoter region was inserted into the pGL3-Basic Luciferase reporter vector (p2000). Four reporter vectors (p2000 + TR13, p2000 + TR14, p2000 + TR15 and p2000 + TR16) were constructed by inserting TRs with four different lengths into the enhancer region of the *ABL1*-promoter vector (p2000) (Fig. 4a). After transfection into 293 T and UC3 cells using these five types of vectors, luciferase activity was investigated. In 293 T cells, the activity of luciferase was significantly reduced when transfecting vectors containing TR alleles of *ABL1*-MS1 compared to when transfected with *ABL1*-promoter vector (p2000) (Fig. 4b). These results suggest that the repeat sequence of *ABL1*-MS1 suppresses the expression of *ABL1*. On the other hand, in the bladder cancer cell line UC3, inhibition of the *ABL1* promoter activity by the repeat sequence was not seen (Fig. 4b).

## Discussion

*ABL1* on chromosome 9 undergoes mutual translocation with the *BCR* gene on chromosome 22 to make the Philadelphia chromosome, which is well known to cause chronic myelogenous leukemia [43–48]. There have been many studies on the breakpoint cluster region in which mutual translocation occurs in the *ABL1*, and it has also been reported that many mutations and repeat sequences exist in the region [2, 4, 49, 50]. Among the repetitive nucleotide sequences found in the human genome, nucleotide sequences such as LINE, SINE, LTR, and SSLP play an important role as a regulator of biological genome evolution and gene expression, and are reported as genetic causes of various diseases [16–20]. Therefore, in this study, the distribution of repetitive sequences within this BCR region was analyzed and a meaningful VNTR region (*ABL1*-MS1) was identified in the intron 1 region of *ABL1*.

In this study, the polymorphism of the *ABL1*-MS1 region was detected, and each *ABL1*-MS1 allele was found to be accurately transmitted from parent to the next generation. In a case–control study using genomic DNA from 207 cancer-free controls and 197 bladder cancer patients, rare alleles (TR14, TR16) with a frequency of less than 1% were identified only in bladder cancer. The rare alleles identified only in bladder cancer showed a statistically significant association with cancer risk (*P* = 0.013). In addition, as a result of comparing the frequency of rare alleles by age based on the age of bladder cancer at 65, there was an increased tendency for association in the younger group (*P* = 0.061). These results suggest that rare *ABL1*-MS1 allele may be genetically related to bladder cancer.

In addition, since abnormal expression of *ABL1* has been reported in solid cancers including bladder cancer [5–8, 26–29], the effect of this VNTR region on the expression of *ABL1* was investigated. First, four TRs (TR13–TR16) identified in this study were sequenced, and analyzed the putative transcription factors using Transfac software. The following transcription factors that appear to be involved in the development and progression of cancer have been identified as being present within the repeat sequence: *GR-alpha*, *GR-beta* [32], *EBF* [33], *C/EBPbeta* [34], *NF-1* [35], *FOXP3* [36], *Pax-5* [37, 38], *p53* [39], *STAT4* [40] and *TFII-I* [41].

Glucocorticoid receptor (GR) is expressed in various isotypes such as *GR-alpha* and *GR-beta*, and it is known as a transcription factor involved in the expression of genes involved in cell cycle arrest and apoptosis, and it has been reported that low expression of GR is related to bladder cancer [32]. Early B-cell factor (*EBF*) is a DNA-binding transcription factor involved in the differentiation and maturation of various cell lineages. It has been reported that deletion of the *EBF* gene contributes to leukemia recurrence and, when inactivated, contributes to tumor formation [33]. CCAAT enhancer binding protein beta (*C/EBP beta*) is involved in various cellular processes and has recently been reported as an essential mediator of breast cancer development [34]. Neurofibromatosis type 1 (*NF-1*) is a tumor suppressor gene that inhibits RAS-GTP activation, and it has recently been reported that *NF-1* mutations are associated with cancer [35]. Forkhead box P3 (*FOXP3*) is a protein involved in the immune system response, and the expression of *FOXP3* has been reported to be associated with poor prognosis in several cancers [36]. The Paired Box-5 (*Pax-5*) gene is a family of nuclear transcription factors [37], has been reported to be expressed in a variety of cancers, and is likely to contribute to overall tumorigenesis [38]. Although *p53* is a tumor suppressor gene and plays an important role in cell death and genomic stability, mutations in the *p53* gene cause cancer [39]. Signal transducer and transcription activator (*STAT4*) play an important role in development, proliferation, and immune defense, but overexpression can lead to several cancers [40]. *TFII-I* was originally a transcription factor capable of binding to two separate promoter elements, a pyrimidine-rich initiator and the recognition site (*E-box*) of upstream factor 1. Recently, it has been reported that it acts as an activator of other factors and affects breast and liver cancer [41]. Therefore, the presence of these transcription factors leads to speculation that the *ABL1*-MS1 region may influence *ABL1* activity.

In order to investigate the effect of the *ABL1*-MS1 region on the expression, four *ABL1*-promoter vectors with TRs inserted into the enhancer region was constructed. When transfected with 293 T, suppression of the luciferase activity by TR was observed, but not in bladder cancer cells. Therefore, this influence on the expression of the TR region suppresses the expression of *ABL1* in non-cancer cells, but does not suppress the expression of *ABL1* in cancer cells. Although we could not find a statistically significant difference based upon TRs length, we identified the potential of *ABL1*-MS1 in *ABL1* expression regulation. We hypothesized that the *ABL1*-MS1 polymorphism loses its ability to suppress the *ABL1* expression during tumor formation.

Our study used a limited number of samples to investigate the effect of repeat sequences in the *ABL1*-MS1 region on control and bladder cancer patients. In the future, studies on samples of various cases of bladder cancer patients are needed, and studies on how the influence of transcription factors related to the *ABL1* promoter region can affect *ABL1*-MS1 in bladder cancer is needed.

## Conclusion

We investigated the structural characterization of the *ABL1* breakpoint region, identified one variable number of tandem repeats (VNTR) present in Intron 1 and named it *ABL1*-MS1. Two rare alleles (TR14, TR16) were detected in bladder cancer, and a statistically significant association was confirmed between the rare *ABL1*-MS1 allele and bladder cancer risk: *P* = 0.013. We also examined the transcription level of the reporter gene driven by the *ABL1* promoter to determine if the VNTR region influenced gene expression, and that VNTR showed inhibition of *ABL1* expression in non-cancer cells 293 T, but not in bladder cancer cells. In addition, it was confirmed that *ABL1*-MS1 is accurately transmitted to offspring according to Mendelian inheritance through meiosis. Therefore, it is suggested that the *ABL1*-MS1 region may affect the regulation of *ABL1* expression in bladder cancer, and the identification of rare alleles of the *ABL1*-MS1 may suggest predictability as a susceptibility marker for bladder cancer.

## Supplementary Information

**Additional file 1**. **Figure S1**. Analysis of repeat sequence distribution of *ABL1* breakpoint cluster region. (**a**) Schematic diagram of repeat sequence within *ABL1* breakpoint region. The black horizontal line represents the *ABL1* breakpoint region and the vertical bar represents the position of the repeat sequence. All repeat sequences were analyzed through Repeatmasker and the UCSC database. (**b**) The diagram represents the number of different repeat sequences within the *ABL1* breakpoint area. **Figure S2**. Analysis of distances between repeat units and identification of putative binding sites for transcription factors of four minisatellites in the *ABL1*-MS1 region. (**a**) Phylogenic trees for the repeat units within each allele of *ABL1*-MS1. Numbers above branches represent bootstrap value (%) for the clades with 1000 replicates. (**b**) Composition of putative transcriptional factors on each minisatellite of the *ABL1*-MS1 region.

**Additional file 2: Supplementary Table S1**. Homology comparison of repeat sequences in the ABL1-MS1 region.

## Data Availability

The structurally analyzed data of the BCR region of *ABL1* can be used in the NCBI database (https://www.ncbi.nlm.nih.gov/gene) and the UCSC genome browser gateway (https://genome.ucsc.edu/cbi-bin/hgGateway). The repeat regions analysis is available in the RepeatMasker (www.repeatmasker.org) and the Tandem repeat finder (http://www.tandem.bu.edu/trf/trf.html). The construction of primers to amplify the selected VNTR regions can be used in the Primer 3 (http://www.bioinfo.ut.ee/primer3-0.4.0/) and the Primer-BLAST (http://www.ncbi.nlm.nih.gov/tools/primer-blast).

## Abbreviations

VNTR: Variable number of tandem repeats
ABL1: Abelson murine leukemia virus oncogene homolog 1
RTK: Receptor tyrosine kinase
cAMP: Cyclic AMP
BCR: Breakpoint cluster region
NMIBC: Non-muscle invasive bladder cancer
MIBC: Muscle invasive bladder cancer
LOH: Loss of heterozygosity
SSLP: Simple sequence length polymorphism
MS: Minisatellite
ORs: Odds ratios
Cls: Confidence intervals
SE: Standard error
SINE: Short interspersed nuclear element
LINE: Long interspersed nuclear element
LTR: Long terminal repeat
GR: Glucocorticoid receptor
EBF: Early B-cell factor
C/EBF beta: CCAAT enhancer-binding protein beta
NF-1: Neurofibromatosis type 1
FOXP3: Forkhead box P3
PAX-5: Paired Box-5
STAT4: Signal transducer and transcription activator 4
E-box: E-recognition site
