# Supplementary figures and images for: VNTR polymorphism in the breakpoint region of ABL1 and susceptibility to bladder cancer
Source: BMC Med Genomics. 2021 May 5;14:121. doi: 10.1186/s12920-021-00968-1 (PMC8097952; doi:10.1186/s12920-021-00968-1)

Additional file 1. Supplementary Figure S1

A

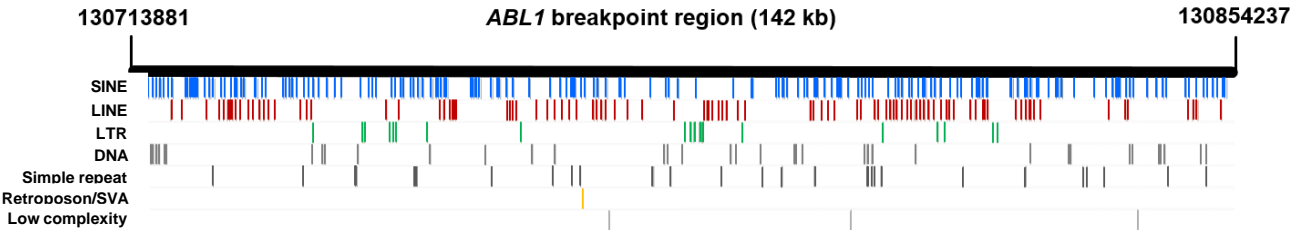

B

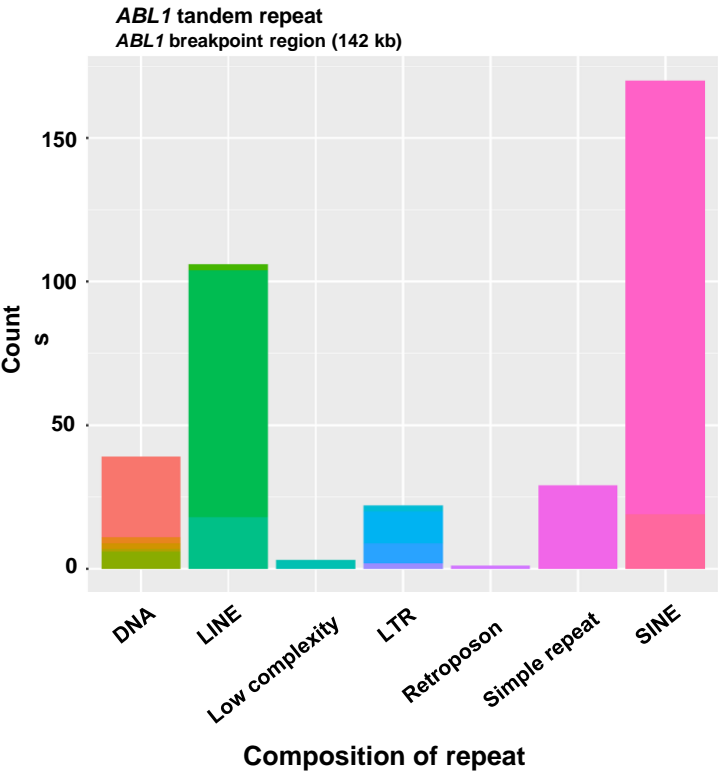

Additional file 1. Supplementary Figure S2

A

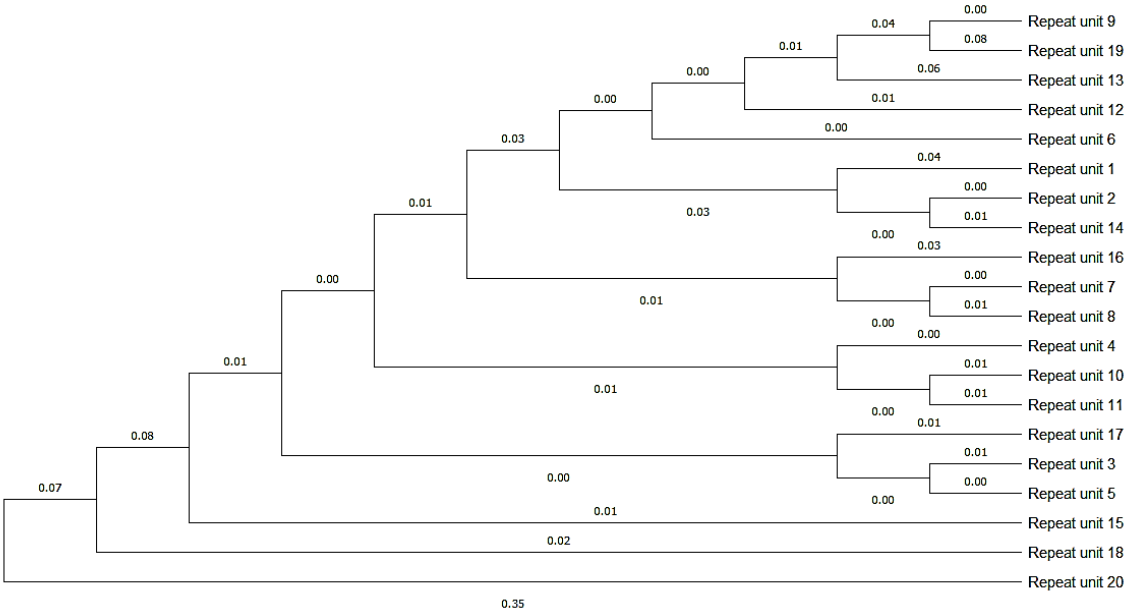

B

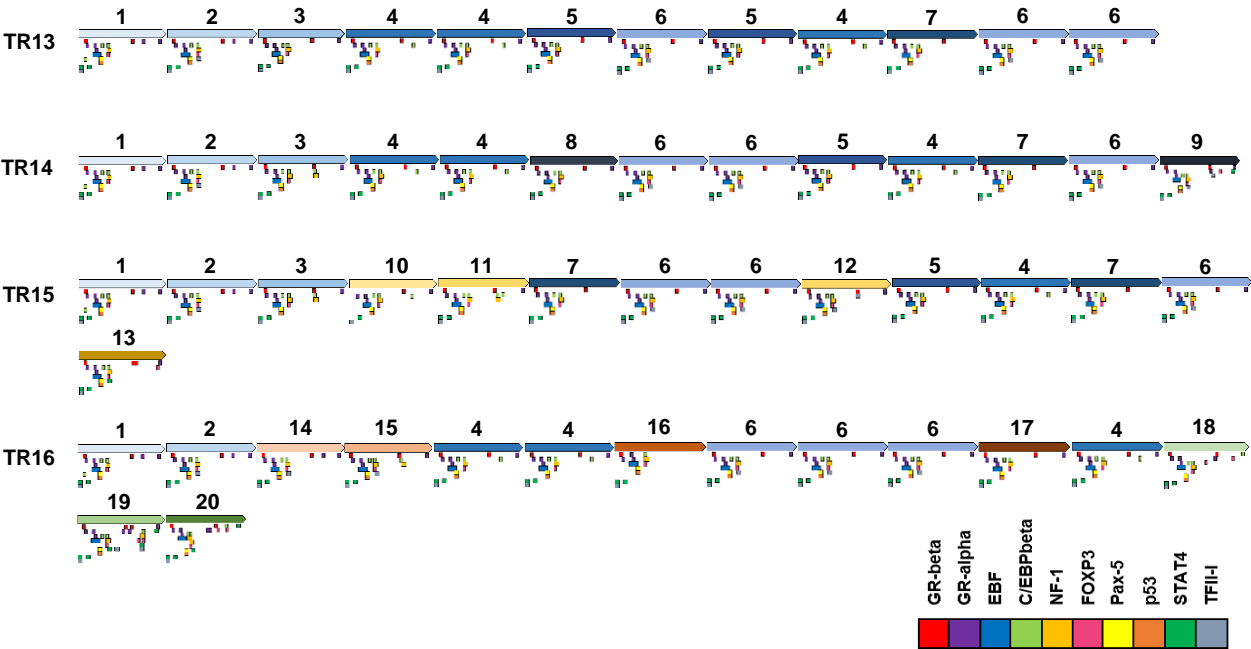

Supplement: Supplementary file 1 — Additional file 1. Figure S1. Analysis of repeat sequence distribution of ABL1 breakpoint cluster region. (a) Schematic diagram of repeat sequence within ABL1 breakpoint region. The black horizontal line represents the ABL1 breakpoint region and the vertical bar represents the position of the repeat sequence. All repeat sequences were analyzed through Repeatmasker and the UCSC database. (b) The diagram represents the number of different repeat sequences within the ABL1 breakpoint area. Figure S2. Analysis of distances between repeat units and identification of putative binding sites for transcription factors of four minisatellites in the ABL1-MS1 region. (a) Phylogenic trees for the repeat units within each allele of ABL1-MS1. Numbers above branches represent bootstrap value (%) for the clades with 1000 replicates. (b) Composition of putative transcriptional factors on each minisatellite of the ABL1-MS1 region. [file 12920_2021_968_MOESM1_ESM.pdf]
